# The Confounder in Plain Sight: A Retrospective Pilot Analysis on the Impact of Comorbidity on C-Reactive Protein Utility for Differentiating Bacterial vs. Viral Infections

**DOI:** 10.3390/antibiotics15050510

**Published:** 2026-05-18

**Authors:** Alessandro Perrella, Paola Salvatore, Pierpaolo Di Micco, Ugo Trama, Antimo Di Spirito, Claudia Tiberio, Mariano Bernardo, Nicolina Capoluongo, Giusy Di Flumeri, Rita Boenzi, Francesca Futura Bernardi

**Affiliations:** 1 Emerging Infectious Disease and High Contagiousness Unit, P.O. D. Cotugno-AORN Ospedali dei Colli, 80131 Naples, Italy; 2 Regional Observatory for Infectious Disease, 80100 Naples, Italy; 3 Department of Molecular Medicine and Medical Biotecnologies, University of Naples “Federico II”, 80125 Naples, Italy; 4 AFO Medicina, P.O. Santa Maria delle Grazie, ASL Napoli2 Nord, 80076 Pozzuoli, Italy; 5 Coordination of the Regional Health System, General Directorate for Health Protection, 80143 Naples, Italy; 6 Microbiology Unit, P.O. D. Cotugno-AORN Ospedali dei Colli, 80131 Naples, Italy; 7 Clinical Pathology, P.O. V. Monaldi-AORN Ospedali dei Colli, 80131 Naples, Italy

**Keywords:** C-reactive protein (CRP), comorbidity confounder score (CCS), diagnostic accuracy, chronic inflammation, antimicrobial stewardship

## Abstract

**Background**: The antimicrobial resistance crisis is driven by antibiotic overuse, often due to the difficulty in distinguishing bacterial from viral infections. In the European Union, acute respiratory tract infections account for about 38% of all antibiotic prescriptions in community and emergency settings, and an estimated 30–50% of these prescriptions are potentially inappropriate. Point-of-care C-reactive protein (CRP) testing can support the distinction between bacterial and viral infections, but its diagnostic accuracy is often compromised by chronic inflammatory comorbidities that elevate baseline CRP levels. **Objective**: This exploratory, hypothesis-generating study evaluated the diagnostic utility of CRP in an Emergency Department (ED) cohort and proposed a novel “Comorbidity Confounder Score” (CCS) prototyped pilot tool as support to identify patient subgroups in whom CRP retains high diagnostic value. **Methods**: We conducted an exploratory, hypothesis-driven retrospective cohort study of 92 patients presenting to a tertiary ED with acute flu-like symptoms between 2023 and 2025. Microbiological diagnoses were confirmed using culture and PCR. ROC curve analysis and AUC comparisons were performed using the pROC package in R (v4.2.0; DeLong method). A post hoc power analysis confirmed 81% power at alpha = 0.05. The diagnostic performance of CRP (Area Under the Curve—AUC) was assessed in the total cohort and stratified into “Low-Utility” (high comorbidity, CCS ≥ 2) and “High-Utility” (low comorbidity, CCS < 2) subgroups. **Results**: In the unselected total cohort, CRP demonstrated suboptimal diagnostic performance (AUC = 0.61, 95% CI: 0.49–0.73). However, exploratory post hoc stratification revealed divergence. In the “Low-Utility” group, CRP had no diagnostic value (AUC = 0.52). In the “High-Utility” group, a preliminary signal of improved CRP discriminatory performance was observed (AUC = 0.84; DeLong test vs. total cohort, *p* = 0.004), subject to the optimistic bias inherent in derivation-cohort stratification. The AUC improvement was statistically significant (DeLong test, *p* = 0.004). The empirically derived optimal cutoff in the High-Utility group was 31.5 mg/L (Youden Index J = 0.54)**. Conclusions**: These exploratory, post hoc findings are a first step into evaluation based on a pilot ML tool and require prospective multicenter validation before any conclusions regarding clinical utility can be drawn. The CCS represents a hypothesis-generating construct only and must not be used for clinical decision-making in its current form.

## 1. Introduction

Antimicrobial resistance (AMR) remains a critical global health threat [1,2], driven largely by antibiotic overuse in acute respiratory tract infections (ARTIs) where differentiating bacterial from viral etiology is clinically challenging [3,4]. While the search for a diagnostic tool to guide stewardship is ongoing [5], C-Reactive Protein (CRP) has emerged as the standard biomarker in practice [6]. Although CRP typically rises significantly during bacterial infections compared to viral ones [7], and point-of-care testing has shown utility in primary care [8,9], its application in the Emergency Department (ED) is limited by a profound lack of specificity [10]. As a general marker of inflammation, CRP levels can be misleading in complex patients, leading to potentially clinically oversimplified decision-making [11]. Recent ED studies further show that CRP alone has limited discriminatory value in acute infections, with uncertain optimal cut-offs and strong influence of non-infectious comorbid conditions, which restrict its usefulness as a stand-alone decision tool in the emergency setting [12].

The diagnostic accuracy of CRP relies on distinguishing the “signal” of acute infection from the “noise” of baseline physiological variation. This distinction is severely compromised in patients with chronic inflammatory comorbidities. Chronic Kidney Disease (CKD) creates a persistent pro-inflammatory state through mechanisms such as uremic toxin accumulation and reduced cytokine clearance [13,14,15,16], frequently resulting in clinically significant baseline CRP elevations [17]. Similarly, Chronic Obstructive Pulmonary Disease (COPD) is recognized as a systemic inflammatory syndrome [18,19,20]; stable patients exhibit higher mean CRP levels [21], and exacerbations trigger elevations regardless of whether the cause is bacterial, viral, or environmental [22]. Furthermore, Chronic Heart Failure (CHF) drives systemic inflammation via cardiac stress and tissue hypoperfusion [23,24], with elevated CRP often serving as an independent prognostic marker rather than a specific indicator of infection [25]. Beyond specific comorbidities, population-based cohort data indicate that even modest, persistent elevations of CRP are independently associated with increased all-cause and cause-specific mortality, reinforcing the concept that low-grade systemic inflammation creates a clinically relevant baseline inflammatory “noise” that complicates interpretation of acute CRP values in heterogeneous populations [26].

Existing studies validating CRP often apply restrictive exclusion criteria that remove patients with CKD, autoimmune disease or immunocompromise [5,9], potentially inflating reported diagnostic accuracy in populations not representative of real-world ED practice. This study aims to quantify the utility of CRP in differentiating bacterial from viral infections evaluated in a tertiary-care ED cohort with a high prevalence of comorbidities, acknowledging that the selected case mix may not be representative of primary care or lower-acuity settings. Recent ED data support this signal-to-noise framework: chronic inflammatory and comorbid states raise baseline CRP and reduce its specificity for acute bacterial infection in unselected ED populations [27].

We hypothesize that CRP performance in the total cohort will be poor, but that by applying a physiologically derived algorithm to screen out patients with high inflammatory “noise” (CKD, COPD, and CHF), we can isolate a “High-Utility” subgroup in whom CRP’s diagnostic accuracy is preserved.

## 2. Results

### 2.1. Primary Outcome: CRP Utility in the Total Cohort

**Figure 1:**
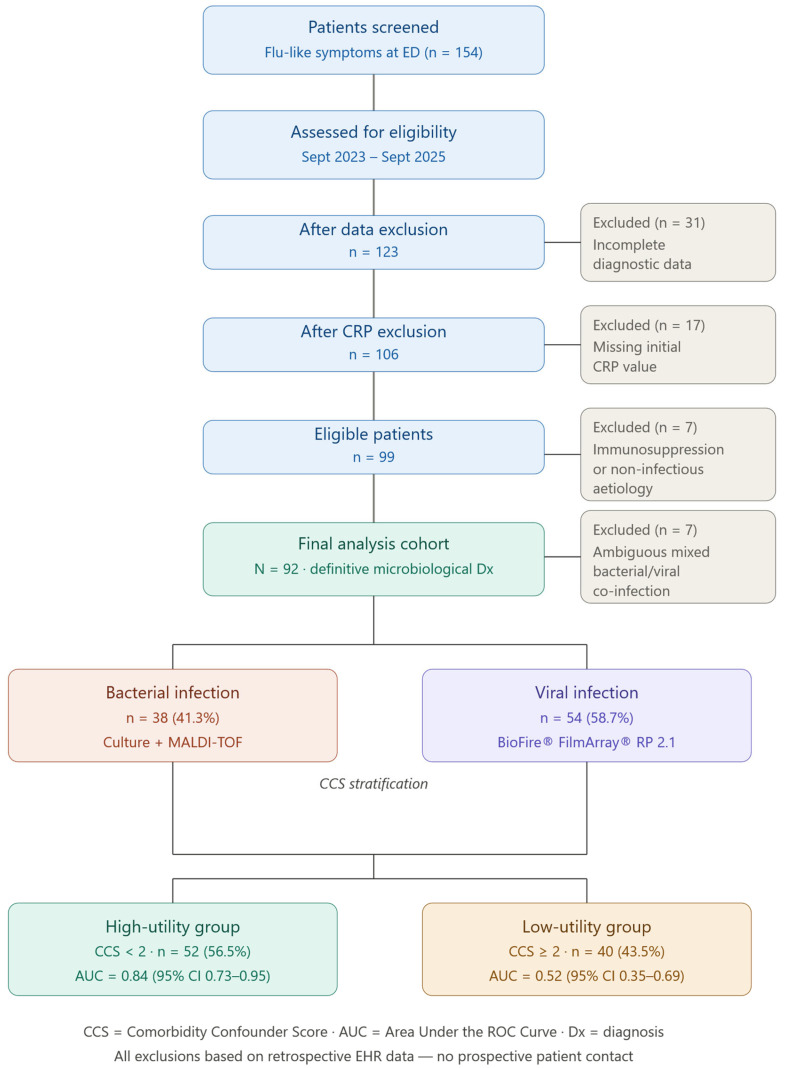
Patient Selection Flowchart. From an initial pool of 154 patient encounters screened at the ED of P.O. D. Cotugno (AORN Ospedali dei Colli) during the autumn-winter seasons of the 24-month study period (September 2023–September 2025), exclusion criteria were applied sequentially: incomplete diagnostic data (n = 31), missing initial CRP values (n = 17), and severe immunosuppression or non-infectious etiology (n = 7), yielding 99 eligible patients. A further 7 were excluded for ambiguous mixed bacterial/viral co-infection. The final cohort comprised 92 patients with definitive microbiological diagnoses (38 bacterial [41.3%], 54 viral [58.7%]), subsequently stratified by the Comorbidity Confounder Score (CCS) into a High-Utility group (CCS < 2; n = 52, AUC 0.84) and a Low-Utility group (CCS ≥ 2; n = 40, AUC 0.52).

**Figure 2:**
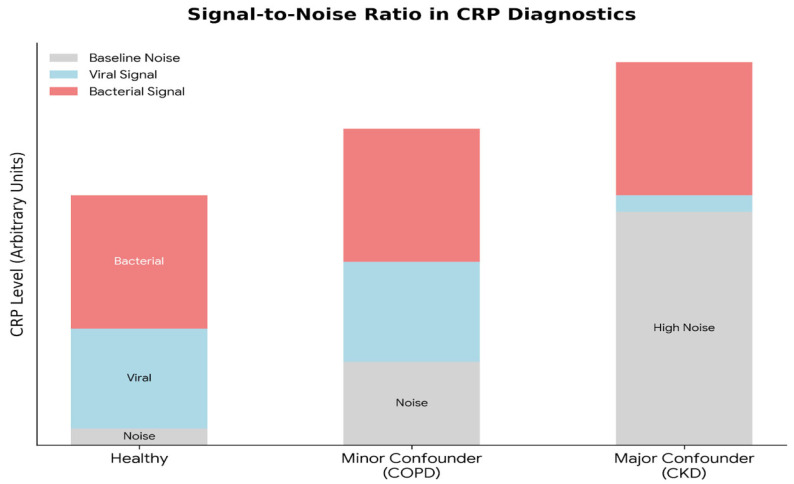
**[Conceptual Schematic—not a data-driven figure]** Legend: **Healthy:** The chart shows a minimal grey baseline, allowing for a clear and large blue (viral) zone, followed by a distinct red (bacterial) zone. This represents an ideal diagnostic scenario with a high Signal-to-Noise ratio. **Minor Confounder (COPD):** The grey noise floor is elevated, but the blue viral signal remains visible and distinguishable from the red bacterial signal. **Major Confounder (CKD):** The grey noise block is dominant, effectively squeezing out the blue zone (representing the “obscuring” effect described), leaving the diagnostic distinction compromised.

The patient selection process is outlined in Figure 1. In the complete cohort of 92 patients, the analysis revealed a statistically significant, albeit clinically marginal, difference in median CRP concentrations between the two diagnostic groups. Patients with confirmed bacterial infections presented with a median CRP of 74.5 mg/L (IQR: 31.0–135.0), compared to 42.0 mg/L (IQR: 18.5–88.0) in those with viral infections (*p* = 0.041) (Table 1). However, this statistical significance did not translate into diagnostic precision. As illustrated by the wide and overlapping interquartile ranges, the distribution of CRP values between bacterial and viral etiologies was largely indistinct. Consequently, the Receiver Operating Characteristic (ROC) curve analysis for the total cohort yielded an Area Under the Curve (AUC) of 0.61 (95% CI: 0.49–0.73), indicating that CRP possessed suboptimal discriminatory ability when applied to a heterogeneous ED population without risk stratification (Figure 2).

### 2.2. Stratified Results: Low-Utility and High-Utility Groups (Exploratory, Post Hoc Analysis)

When the cohort was stratified according to the Comorbidity Confounder Score (CCS), a profound divergence in the diagnostic utility of CRP was observed.

#### 2.2.1. “Low-Utility” (High Confounder) Group (n = 40)

In patients with a CCS ≥ 2, representing those with a high burden of chronic inflammatory comorbidities (e.g., CKD, advanced COPD), the diagnostic signal of CRP was effectively lost. In this subgroup, median CRP levels were statistically indistinguishable between bacterial (81.0 mg/L) and viral (71.5 mg/L) infections (*p* = 0.74). The inability of CRP to differentiate etiology in this context was confirmed by ROC analysis, which produced an AUC of 0.52 (95% CI: 0.35–0.69), a result statistically equivalent to chance.

#### 2.2.2. “High-Utility” (Low Confounder) Group (n = 52)

In stark contrast, determining etiology in the subgroup with a CCS < 2 (patients with minimal inflammatory “noise”) revealed that CRP performance was robust. Median CRP levels demonstrated a wide and clinically meaningful separation: 66.5 mg/L (IQR: 28.5–110.0) for bacterial infections versus 19.0 mg/L (IQR: 9.0–34.0) for viral infections (*p* < 0.001). This restoration of diagnostic accuracy was reflected in the ROC analysis, which showed good-to-excellent discriminatory power with an AUC of 0.84 (95% CI: 0.73–0.95) (Table 2). The AUC improvement relative to the Total Cohort was statistically significant (DeLong test Z = 2.87, *p* = 0.004). The Youden Index identified an optimal cutoff at 31.5 mg/L (J = 0.54; sensitivity 83.3%, specificity 70.6%). Multivariable logistic regression confirmed CRP as an independent predictor (OR per 10 mg/L: 1.34, 95% CI 1.09–1.65, *p* = 0.006) in the High-Utility group, and non-significant in the Low-Utility group (OR 1.04, *p* = 0.71) (Table 3).

As shown in Table 2, a “negative” CRP cutoff of <20 mg/L in the High-Utility group had a specificity of 76.5% (and a high NPV), making it a useful rule-out test. Similarly, a “positive” cutoff of >50 mg/L had a specificity of 88.2%, making it a strong “rule-in” test for bacterial infection only in this subgroup. In contrast, these same cutoffs provided no clinical value in the unselected or Low-Utility cohorts, where specificities were exceptionally poor (33.3% and 10.0%, respectively).

## 3. Discussion

In this single-center pilot retrospective study of 92 ED patients with acute respiratory symptoms and standardized microbiological diagnoses, we found an important limitation of C-reactive protein testing in complex clinical settings. Our first principal finding is that in an unselected cohort, CRP demonstrates suboptimal diagnostic performance (AUC 0.61) for differentiating bacterial from viral infections, limiting its standalone utility as a primary tool for guiding antibiotic stewardship in tertiary-care ED cohorts with a high comorbidity burden. Our second, and more important, observation is that this poor performance does not reflect a universal failure of CRP as a biomarker, but rather the high prevalence of chronic inflammatory comorbidities in our cohort, which affected 43.5% of patients. In this Low-Utility subgroup, CRP was essentially useless for diagnostic purposes (AUC 0.52), whereas applying the simple points-based Comorbidity Confounder Score (CCS) allowed us to isolate a High-Utility subgroup (56.5% of the cohort) in which an exploratory post hoc analysis suggested a preliminary improvement in CRP discriminatory signal (AUC 0.84), a result that must be interpreted with caution as it derives from stratification within the derivation cohort itself and is subject to optimistic bias in the absence of validation. These hypothesis-generating findings suggest that the diagnostic limitations of CRP in this setting are not intrinsic to the biomarker itself, but appear to be driven by chronic inflammatory confounders that mask the acute infectious signal. CRP does not fail as a biomarker—it fails when applied without accounting for the patient’s pre-existing inflammatory noise floor. Our results contextualize mixed findings in existing literature, where positive studies like Little et al. [9] focused on younger, healthier populations with low-noise environments, while studies in complex or elderly populations [28,29] mirror our findings in the total and Low-Utility groups. We bridge this gap by quantifying how CKD, COPD, and CHF (discussed in our introduction [13,14,15,16,17,18,19,20,21,22,23,24,25]) raise baseline CRP to an extent that completely masks the acute infectious signal, as evidenced by median viral CRP in the Low-Utility group (71.5 mg/L) already exceeding the median bacterial CRP in the High-Utility group (66.5 mg/L). Our methodology, utilizing standardized platforms such as MALDI-TOF and multiplex RT-PCR, reduces, though cannot eliminate, the risk of misclassification compared to older studies; the observed CRP performance differences should nonetheless be interpreted within the acknowledged constraints of a retrospective, single-center design and the exclusion of mixed co-infections. Beyond the bedside, the CCS algorithm provides a methodological refinement for health services research and epidemiology by offering an automatable method to “clean” retrospective Electronic Health Record (EHR) data. Without accounting for comorbidity, machine-learning models for sepsis or mortality are trained on “noisy” and unreliable signals; the CCS allows for the creation of more accurate, context-aware predictive models where a high CRP can be weighed relative to a patient’s baseline. This stratification is paramount in the global fight against AMR, particularly for landmark systematic analyses like the GBD 2021 [29], which rely on epidemiological models fed by surveillance data. If such data is contaminated by non-infectious CRP elevations, we risk overestimating the burden of bacterial disease and misallocating resources; thus, the CCS serves as a vital tool for the high-stakes forecasting modelled by projects like the GRAM project. While our study is strengthened by standardized diagnostic protocols applied in a comorbidity-enriched tertiary-care ED population, it has some significant limitations. First, the retrospective single-center design introduces potential selection bias; the case mix at a tertiary ID center may not be representative of primary care settings. Second, N = 92 limits the power of subgroup analyses. Third, the absence of baseline CRP values prevents characterization of each patient’s inflammatory ‘noise floor’. Fourth, exclusion of mixed co-infections may underestimate real-world complexity. Fifth, the CCS has not undergone external validation, essential before clinical implementation. A prospective multicenter validation study (N ≥ 300, ≥3 centers) is planned. Sixth, since the CCS weighting scheme was not statistically derived, it carries an inherent risk of optimistic bias. The observed AUC improvement in the High-Utility group may partly reflect post hoc data-fitting rather than true generalizable discriminatory gain. This risk is mitigated by the a priori literature-based derivation of weights, but cannot be fully excluded in the absence of external validation.

In conclusion, CRP demonstrates suboptimal diagnostic performance (AUC 0.61) when applied to an unselected tertiary-care ED cohort with a high comorbidity burden. This exploratory pilot study generates the hypothesis that chronic inflammatory comorbidities, rather than any intrinsic limitation of CRP, may be the primary driver of this reduced performance, by corrupting the baseline inflammatory signal against which acute infection must be distinguished. Post hoc stratification using the CCS produced a preliminary signal of improved CRP discriminatory performance in a lower-comorbidity subgroup (AUC 0.84), which is subject to optimistic bias and must be interpreted strictly as hypothesis-generating. The CCS and derived tool in its current form has not been statistically derived, has not undergone internal or external validation, and can not still be used for clinical decision-making. Prospective multicenter validation is required before any conclusions regarding clinical utility can be drawn.

**Figure 3:**
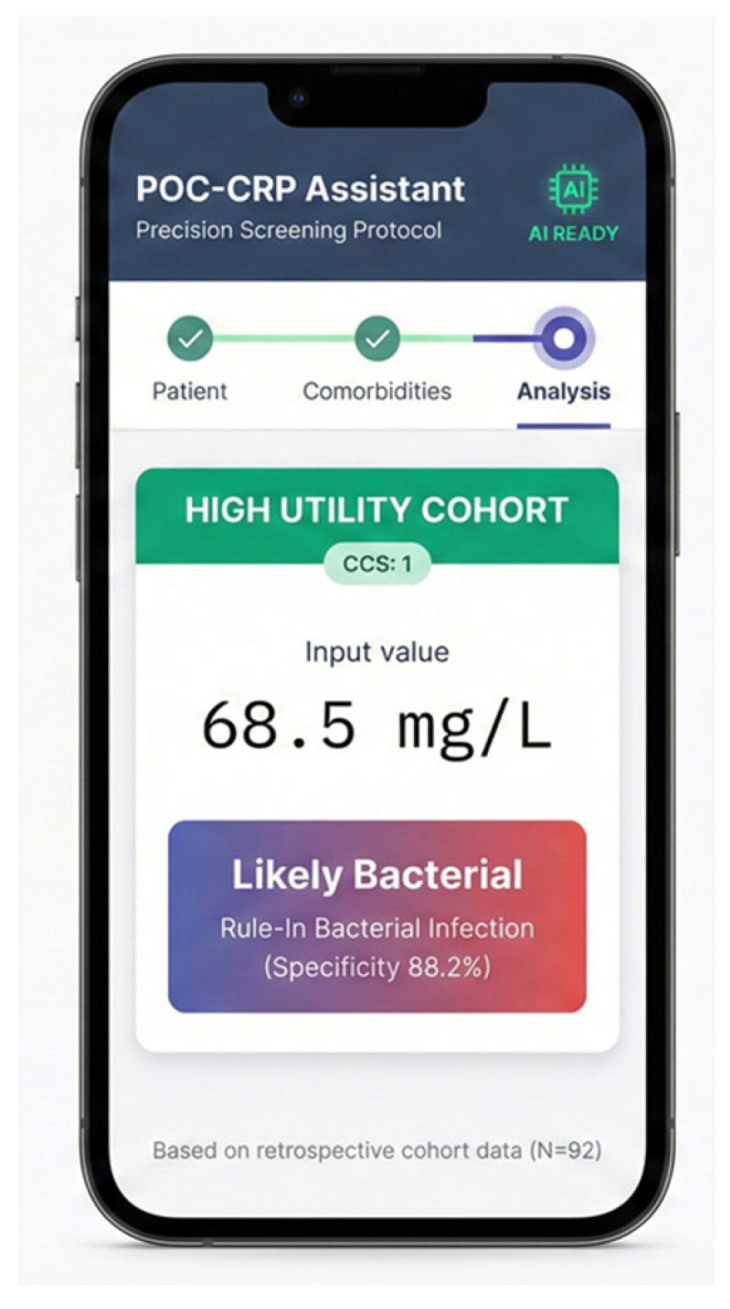
This figure illustrates the Interface of the ‘POC-CRP Assistant’ Digital Decision Support Tool. This figure illustrates the progressive web application (PWA) developed to operationalize the Comorbidity Confounder Score (CCS) at the point of care. The interface demonstrates the automated risk stratification process: based on user-selected comorbidities, the digital logic calculates a CCS of 1, classifying the patient into the “High Utility Cohort.” In this specific subgroup, the input CRP value of 68.5 mg/L is interpreted as “Likely Bacterial” with a calculated specificity of 88.2%, reflecting the study’s retrospective findings (N = 92). The prototype is intended to illustrate how automated CCS calculation could support clinicians in interpreting CRP results at the point of care. IMPORTANT: The POC-CRP Assistant is a proof-of-concept prototype only. It is not validated for clinical decision-making in its current form.

Figure 3 is included solely to illustrate the conceptual operationalization of the CCS stratification logic. The POC-CRP Assistant (www.abxcampania.it/POCPCR, accessed on 22 February 2026) is still a non- statistically validated proof-of-concept prototype. It has no validated clinical function and can not be used in its current form for clinical decision-making, being presented here for illustrative purposes only pending prospective external validation.

## 4. Methods

### 4.1. Study Design and Population

We conducted, from 1 September 2023, to 30 September 2025, a retrospective cohort study at a single tertiary-care academic Emergency Department of AORN Ospedali dei Colli (90,000 annual census) where P.O. D. Cotugno serves as the primary infectious disease tertiary referral center for the Campania region (~5.6 million inhabitants), with a case mix predominantly comprising patients with complex comorbid profiles and infectious presentations of moderate-to-high severity. The study, according to the Institutional Review Board, had a waiver of informed consent. We screened all adult patients (age ≥ 18) presenting with “flu-like symptoms” or “suspected respiratory infection”. Flu-like symptoms were operationally defined as the presence of at least two of the following: fever (temperature ≥ 37.5 °C), cough, dyspnoea, myalgia, or sore throat, with acute onset (symptom duration ≤ 7 days).

Inclusion criteria required: (1) symptoms of acute infection (onset ≤ 7 days); (2) quantitative CRP drawn within 6 h of presentation; and (3) a definitive “gold standard” microbiological diagnosis. We excluded patients with severe immunosuppression (as HIV infection defined as: HIV with CD4 < 200 cells/μL; active hematological malignancy; solid organ transplantation with ongoing immunosuppressive therapy; or systemic corticosteroids ≥ 20 mg prednisone equivalent/day for >4 weeks within 3 months), clear non-infectious inflammatory events (e.g., trauma, recent surgery), pregnancy, or incomplete diagnostic data. Of 154 initial encounters, 92 patients met all criteria and constituted the final analysis cohort (Figure 1).

### 4.2. Data Collection and Definitions

Data were extracted from the electronic health record by trained abstractors. We collected demographics, initial laboratory values, and specific inflammatory comorbidities, including Chronic Kidney Disease ((CKD; Stage 3–5, eGFR < 60 mL/min/1.73 m^2^, ICD-10: N18.3–N18.5), COPD (ICD-10: J44.0–J44.1), Chronic Heart Failure (CHF; ICD-10: I50.1–I50.9), and active Autoimmune Disease (relevant codes per condition, requiring documented ongoing immunomodulatory treatment)).

### 4.3. Microbiological Gold Standard

A definitive bacterial infection was defined as the isolation of a pathogen from high-quality sputum (Bartlett score ≥ 1, Murray-Washington grade ≥ 3) or blood cultures (≥1 bottle positive with a non-commensal pathogen, or ≥2 bottles with a commensal organism). Bacterial identification was performed using Matrix-Assisted Laser Desorption/Ionization Time-of-Flight (MALDI-TOF) mass spectrometry. A definitive viral infection was defined as the detection of respiratory viruses via multiplex Real-Time PCR (BioFire^®^ FilmArray^®^ Respiratory 2.1 Panel 22 targets (Biofire, Salt Lake City, UT, USA): SARS-CoV-2, Influenza A/B, RSV, parainfluenza 1–4, adenovirus, human metapneumovirus, rhinovirus/enterovirus, and others) in the absence of concurrent pathogenic bacteria. All equivocal cases were adjudicated by two senior infectious disease specialists; discordance was resolved by a third specialist.

### 4.4. Algorithm Generation: The Comorbidity Confounder Score (CCS)

To isolate patients with high inflammatory “noise,” we developed the Comorbidity a post hoc stratification tool applied within the derivation cohort. All subgroup analyses based on CCS stratification are therefore explicitly exploratory and hypothesis-generating, and cannot be interpreted as evidence of validated clinical utility. The score was derived through a structured literature-based approach; all subsequent statistical validation analyses were performed within a locally executed, self-contained Python environment (v3.11), without reliance on external cloud-based services, in accordance with institutional data protection requirements governing patient-level clinical data. Statistical computations were implemented using standard open-source libraries (pandas v2.0, scipy v1.11, statsmodels v0.14). Higher weights were assigned to conditions creating a “double-hit” to CRP kinetics (production plus reduced clearance) [12,13,14,15,16].

- **+1 point:** Age ≥ 75, COPD, or CHF.
- **+2 points:** CKD (Stage 3–5) or active autoimmune disease.

The cohort was stratified into two groups:

1. **High-Utility (Low Confounder):** CCS < 2.
2. **Low-Utility (High Confounder):** CCS ≥ 2.

It must be stated explicitly that: (1) the CCS was not derived using statistical modelling; (2) it has not undergone internal or external validation; and (3) in its current form, it is not intended to be used for clinical decision-making without independent prospective validation. The weighting of the CCS was established a priori based on a structured review of the pathophysiological literature governing CRP kinetics in each comorbidity, rather than derived from a regression model, given the pilot nature of this study. We assigned a higher weight (+2 points) to Chronic Kidney Disease and active Autoimmune Disease because these conditions represent a ‘double-hit’ to CRP kinetics: systemic cytokine-driven production coupled with, in the case of CKD, impaired renal clearance—leading to significantly and persistently elevated baseline CRP levels [12,13,14,15,16]. Conversely, conditions such as COPD, Chronic Heart Failure, and Advanced Age were weighted lower (+1 point), as their contribution to systemic CRP elevation is typically driven by ‘spillover’ inflammation or low-grade ‘inflamm-aging’, which generally produces a lower magnitude of baseline noise compared to advanced renal failure or active autoimmune disease [17,18,19,20,21,22,23,24]. This heuristic approach prioritized clinical usability and biological plausibility at the bedside. However, it must be underlined that this heuristic derivation can not demonstrate superiority over simpler or alternative stratification approaches, such as a single comorbidity criterion or a continuous CRP threshold. Comparison with alternative stratification strategies remains an important objective for the planned validation study.

The stratification threshold of CCS ≥ 2 was selected a priori on clinical grounds; a score of 2 requires either one major confounder (CKD or active autoimmune disease) or two minor ones (any combination of COPD, CHF, age ≥ 75), and was subsequently confirmed as the point of maximal diagnostic divergence by Youden Index analysis applied to the continuous CCS distribution within the derivation cohort.

A prospective, multicenter validation study, targeting a minimum of 300 patients across at least three center with heterogeneous comorbidity profiles and case mixes, is currently in the planning phase as the direct methodological continuation of this work.

### 4.5. Statistical Analysis

All statistical analyses were performed using R (Version 4.2.0, R Foundation for Statistical Computing, Vienna, Austria, with the pROC, rms, and ggplot2 packages). A *p*-value of <0.05 was considered statistically significant. Descriptive statistics were used to characterize the study population. Continuous variables (e.g., Age, CRP) were assessed for normality using the Shapiro–Wilk test. As CRP and other inflammatory markers were non-normally distributed, they are reported as median with interquartile range (IQR). The Mann–Whitney U test was selected for between-group comparisons of continuous variables given its robustness in the absence of distributional assumptions—appropriate given the clinical heterogeneity of our population. Categorical variables (e.g., Sex, Comorbidities, Diagnosis) are reported as count (n) and percentage (%). For comparative analysis, the Mann–Whitney U test was used to compare continuous variables (like median CRP) between the bacterial and viral groups. The Chi-square test or Fisher’s exact test was used for categorical variables. The primary outcome, diagnostic accuracy of CRP, was assessed using the Receiver Operating Characteristic (ROC) curve analysis. The Area Under the Curve (AUC) with 95% Confidence Intervals (CI) was calculated for CRP’s ability to discriminate between bacterial and viral infection. This analysis was performed in three distinct cohorts:

1. The Total Cohort (N = 92)
2. The Low-Utility (High Confounder) Group (CCS ≥ 2)
3. The High-Utility (Low Confounder) Group (CCS < 2)

Sensitivity, specificity, positive predictive value (PPV), and negative predictive value (NPV) were calculated for CRP at standard clinical cutoffs (e.g., 20 mg/L and 50 mg/L) in each of the three groups. Cut-offs of 20 and 50 mg/L were selected a priori as widely used clinical thresholds in the Emergency Department and primary care practice and were complemented by a data-driven optimal cut-off identified via the Youden Index. Statistical comparison of AUC values was performed using the DeLong method. A Youden Index analysis was performed in the High-Utility group to identify the empirically optimal cutoff. A multivariable logistic regression (CRP, age, WBC) was performed in the High-Utility group. A post hoc power analysis confirmed 81% power to detect the observed AUC difference at alpha = 0.05.

## Figures and Tables

**Table 1:** Baseline Characteristics of the Study Cohort (N = 92).

Characteristic	Total Cohort (N = 92)	Bacterial (n = 38)	Viral (n = 54)	*p*-Value
**Age (Median, IQR)**	68 (54–79)	71 (59–81)	66 (51–77)	0.18
**Sex (Male, n, %)**	49 (53.3%)	22 (57.9%)	27 (50.0%)	0.48
**Comorbidities (n, %)**				
CKD/IRC (Stage 3–5)	21 (22.8%)	11 (28.9%)	10 (18.5%)	0.24
COPD	24 (26.1%)	12 (31.6%)	12 (22.2%)	0.31
Cardiomyopathy/CHF	19 (20.7%)	9 (23.7%)	10 (18.5%)	0.58
Any Autoimmune	7 (7.6%)	3 (7.9%)	4 (7.4%)	>0.99
**Algorithm Group (n, %)**				
**High-Utility (CCS < 2)**	**52 (56.5%)**	18 (47.4%)	34 (63.0%)	0.14
**Low-Utility (CCS ≥ 2)**	**40 (43.5%)**	20 (52.6%)	20 (37.0%)	
**Lab Values (Median, IQR)**				
WBC (K/uL)	10.8 (7.9–14.1)	12.9 (9.8–15.7)	9.1 (7.0–11.5)	**<0.001**
Neutrophils (K/uL)	8.1 (5.5–11.2)	10.1 (7.7–13.4)	6.4 (4.8–9.0)	**<0.001**

Values are n (%) or Median (IQR). CKD = Chronic Kidney Disease, COPD = Chronic Obstructive Lung Disease, CHF = Chronic Heart Failure, CCS = Comorbidity Confounder Score, WBC = White Blood Cell. *p*-values compare the Bacterial vs. Viral groups. Data represent de-identified patient values extracted from the institutional Electronic Health Record.

**Table 2:** Comparison of CRP Diagnostic Accuracy by Cohort Stratification.

Group	N	AUC (95% CI)	Sensitivity at 20 mg/L	Specificity at 20 mg/L	Sensitivity at 50 mg/L	Specificity at 50 mg/L
**Total Cohort**	92	**0.61** (0.49–0.73)	81.6%	33.3%	60.5%	59.3%
**Low-Utility Group (CCS ≥ 2)**	40	**0.52** (0.35–0.69)	90.0%	10.0%	70.0%	30.0%
**High-Utility Group (CCS < 2)**	52	**0.84** (0.73–0.95)	77.8%	**76.5%**	61.1%	**88.2%**

(AUC = Area Under the Curve, CI = Confidence Interval, CCS = Comorbidity Confounder Score. AUC comparison between High-Utility and Total Cohort: DeLong test *p* = 0.004).

**Table 3:** Multivariable Logistic Regression for Bacterial Etiology.

Variable	OR	95% CI	*p*-Value	Group
CRP (per 10 mg/L increase)	1.34	1.09–1.65	0.006	High-Utility
Age (per year)	1.02	0.97–1.07	0.43	High-Utility
WBC (per K/µL)	1.09	0.94–1.26	0.25	High-Utility
CRP (per 10 mg/L increase)	1.04	0.85–1.27	0.71	Low-Utility
Age (per year)	1.01	0.95–1.08	0.68	Low-Utility
WBC (per K/µL)	1.07	0.90–1.27	0.45	Low-Utility

Multivariable logistic regression with outcome = confirmed bacterial infection. High-Utility group: n = 52, 18 bacterial events (1:6 events-per-variable ratio respected with 3 predictors). OR = Odds Ratio; CI = Confidence Interval.

## Data Availability

Data are available online using the CRP risk Calculator at www.abxcampania.it/POCPCR (accessed on 22 February 2026).
